# Selective Inhibition of *Helicobacter pylori* Carbonic Anhydrases by Carvacrol and Thymol Could Impair Biofilm Production and the Release of Outer Membrane Vesicles

**DOI:** 10.3390/ijms222111583

**Published:** 2021-10-27

**Authors:** Rossella Grande, Simone Carradori, Valentina Puca, Irene Vitale, Andrea Angeli, Alessio Nocentini, Alessandro Bonardi, Paola Gratteri, Paola Lanuti, Giuseppina Bologna, Pasquale Simeone, Clemente Capasso, Viviana De Luca, Claudiu T. Supuran

**Affiliations:** 1 Department of Pharmacy, “G. d’Annunzio” University of Chieti-Pescara, 66100 Chieti, Italy; rossella.grande@unich.it (R.G.); valentina.puca@unich.it (V.P.); irene.vitale@unich.it (I.V.); 2 Neurofarba Department, University of Florence, 50019 Sesto Fiorentino, Italy; andrea.angeli@unifi.it (A.A.); claudiu.supuran@unifi.it (C.T.S.); 3 Laboratory of Molecular Modeling Cheminformatics & QSAR, Section of Pharmaceutical and Nutraceutical Sciences, Neurofarba Department, University of Florence, 50019 Sesto Fiorentino, Italy; alessio.nocentini@unifi.it (A.N.); alessandro.bonardi@unifi.it (A.B.); paola.gratteri@unifi.it (P.G.); 4 Department of Medicine and Aging Science, “G. d’Annunzio” University of Chieti-Pescara, 66100 Chieti, Italy; paola.lanuti@unich.it (P.L.); giuseppina.bologna@hotmail.it (G.B.); pasquale.simeone@unich.it (P.S.); 5 Center for Advanced Studies and Technology (CAST), University “G. d’Annunzio” of Chieti-Pescara, 66100 Chieti, Italy; 6 Department of Biology, Agriculture and Food Sciences, National Research Council (CNR), Institute of Biosciences and Bioresources, 80131 Naples, Italy; clemente.capasso@ibbr.cnr.it (C.C.); viviana.deluca@ibbr.cnr.it (V.D.L.)

**Keywords:** carvacrol, thymol, *Helicobacter pylori*, carbonic anhydrase, anti-biofilm activity, molecular modelling, amoxicillin, probiotic bacteria

## Abstract

*Helicobacter pylori*, a Gram-negative neutrophilic pathogen, is the cause of chronic gastritis, peptic ulcers, and gastric cancer in humans. Current therapeutic regimens suffer from an emerging bacterial resistance rate and poor patience compliance. To improve the discovery of compounds targeting bacterial alternative enzymes or essential pathways such as carbonic anhydrases (CAs), we assessed the anti-*H. pylori* activity of thymol and carvacrol in terms of CA inhibition, isoform selectivity, growth impairment, biofilm production, and release of associated outer membrane vesicles-eDNA. The microbiological results were correlated by the evaluation in vitro of *H. pylori* CA inhibition, in silico analysis of the structural requirements to display such isoform selectivity, and the assessment of their limited toxicity against three probiotic species with respect to amoxicillin. Carvacrol and thymol could thus be considered as new lead compounds as alternative *H. pylori* CA inhibitors or to be used in association with current drugs for the management of *H. pylori* infection and limiting the spread of antibiotic resistance.

## 1. Introduction

Recent metadata analyses have described antimicrobial resistance as an evolving global threat to the human population and provided alarming estimates for the near future. The World Health Organization (WHO) constantly establishes antibiotic resistance surveillance campaigns to avoid misuse/abuse of antibiotics, stimulate the discovery of new antibiotics characterized by alternative mechanisms of action, validate bacterial protein/enzymes as novel targets to be explored, and limit the resistance phenomena ascribed to the clinically used antimicrobial arsenal [1]. In more detail, in 2017, the WHO claimed an urgent need for novel therapies against twelve high priority pathogens such as *Helicobacter pylori* by listing clarithromycin-resistant strains to be addressed for antimicrobial research development. Beyond the large plethora of synthesized compounds, natural compounds can be biologically and chemically studied to bring to light bioactive products endowed with beneficial biological activity [2,3].

In such a scenario, pathogenic and non-pathogenic carbonic anhydrase (CA, EC 4.2.1.1)-expressing bacteria are of particular interest due to the underexplored structural differences among bacterial α-, β-, γ-, and ι-CAs and other isoforms encoded by other species (especially mammal homologues) [4]. These enzymes, catalyzing the pivotal CO_2_ hydration equilibrium, are important for sustaining and modulating metabolism and virulence of microorganisms. It has been recently demonstrated that carbonic anhydrase inhibition led to impaired bacterial growth (bacteriostatic or bactericidal effects), reduced expression of virulence factors, and furnished an alternative option in combination with the current therapeutically used drugs [5]. This strategy has been applied to different Gram-positive and Gram-negative bacterial species (*Escherichia coli*, *H. pylori*, *Mycobacterium tuberculosis*, *Vibrio cholerae*, *Pseudomonas aeruginosa*, *Staphylococcus aureus*, *Porphyromonas gingivalis*, *Streptococcus* spp., *Enterococcus* spp., etc.), hampering their ability to develop resistance to common antimicrobials and leading to innovative drug combinations to overcome the spreading of resistant strains [6,7,8,9,10,11,12,13].

In detail, *H. pylori* CAs help to modulate pH homeostasis (as a result of a complex interplay with urease) in order to survive in an overly acidic gastric environment. *H. pylori* genome encodes for two CAs (HpCAα and HpCAβ) characterized by different subcellular localization (periplasm and cytoplasm, respectively) and inhibitor selectivity [14]. Their expression, stimulated under acidic conditions and induced by the two-component system known as ArsRS, can influence the buffering potential to ensure proper colonization and survival. More intriguingly, *H. pylori* CAs are strictly related to bacterial membrane integrity and the ability to produce biofilm [14]. These proteins can thus be considered druggable targets in these prokaryotes with limited toxicity to human cells.

*H. pylori* infections account for almost half of the global population, with an alarming trend of recurrence and lack of eradication after therapy. If untreated or not correctly cured, this nasty pathogen induces gastric inflammatory responses developing into pathological conditions (gastritis, peptic ulcer, gastric cancer, mucosa-associated lymphoid tissue) during its adaptation and replication within the stomach environment. In addition, the current pharmacological therapy is hampered by the development of resistance mechanisms, making the available arsenal the last-resort medicines [15].

The capability of *H. pylori* to produce biofilm represents a survival strategy adopted by the microorganism to protect itself by both the host immune system and the attack of antimicrobials [16]. The tridimensional structure of *H. pylori* biofilm is mainly represented by cells surrounded by a self-produced extracellular polymeric substances (EPS) matrix composed by proteomannans, lipopolysaccharide-related structures, extracellular DNA (eDNA), proteins, and outer membrane vesicles (OMVs). The OMVs are heterogeneous bilayer structures, 50–250 nm in diameter, delivering chemically different macromolecules (i.e., phospholipids, proteins, lipopolysaccharide (LPS), and periplasmic components) [17,18]. Although few data are available about the biological role of OMVs, several studies have demonstrated that OMVs are involved in virulence factors and DNA transfer as well as in signaling among bacteria [19]. It has been demonstrated that OMVs contribute to a direct eDNA-mediated cell–cell binding inside the biofilm, suggesting that they play a role in biofilm development and stability [20,21]. In addition, OMVs protect the eDNA by nuclease degradation, promoting the horizontal gene transfer mechanism, which is surely favored by the biofilm niche [21,22]. We have recently demonstrated the presence of α-CA in the OMVs produced by *H. pylori* in both planktonic (pOMVs) and biofilm (bOMVs) phenotypes [23]. These data suggest that the inhibition of α-CA interferes with the microorganism colonization and survival as well as with *H. pylori* pathogenesis merely destabilizing the production of OMVs [23].

The first examples of *H. pylori* CA inhibitors took advantage of the structures of well-established drugs also acting on human CAs [24,25]. Conversely, among the scientific studies dealing with the anti-*H. pylori* of natural products, we have demonstrated that carvacrol and thymol can inhibit the growth of several reference and clinical *H. pylori* strains (MIC range 16–64 μg/mL and 64–128 μg/mL, respectively) and that modifications of the chemical structure could lead to more potent inhibitors [26,27]. Focusing on the specific mechanism of action of the parent compounds [28,29,30] and on the possibility to further limit the biofilm produced by the pathogen, we decided to better explore if these two naturally occurring compounds could inhibit in vitro and in silico the two *H. pylori* CAs and how this inhibition would impact other microbiological aspects (biofilm inhibition, outer membrane vesicles production, associated eDNA content) with respect to amoxicillin as a reference drug.

Furthermore, the effect of new antimicrobial molecules on the human microbiota is rarely determined; therefore, the discovery of new molecules/compounds, characterized by a selective toxicity between pathogens and components of human microbiota, might represent a successful strategy to fight microbial infections [31]. Keeping in mind the contribution of the gut microbiome to human health and protection against invasive pathogens [32], we aimed at exploring the effects of carvacrol and thymol against three probiotic species selected as a control, encoding for α- or γ-CA, to further assess isoform selectivity.

## 2. Results

### 2.1. CA Inhibition Data and Selectivity Profiling

For comparison, the two compounds were evaluated for the inhibition properties against the human-expressed (h) CA I, II, and VI isoforms (belonging to the α family from *Homo sapiens*); the two CAs cloned and purified from *H. pylori* (HpCAα and HpCAβ); the other bacterial PgiCAβ (from *Porphyromonas gingivalis*) and SmuCA (from *Streptococcus mutans*); and the fungal β-CA (from *Malassezia globosa*) by means of the stopped-flow technique applied to the CO_2_ (carbon dioxide) hydrase assay [33]. The inhibition data, compared to those of the standard sulphonamide inhibitor acetazolamide (AAZ), are reported in Table 1.

Differently from acetazolamide, well-known to be a potent albeit pan-isoform α- and β-CA inhibitor with the possibility to induce unwanted side effects, carvacrol and thymol displayed any appreciable inhibition of human α-CAs (*K*_I_ >100 μM against hCA I, II, and VI). Towards *H. pylori* CAs, these natural regioisomers presented a peculiar trend and a promising inhibitory activity, acting as competitive inhibitors mimicking the transition state of the reaction. Carvacrol was equipotent against both HpCAs in the micromolar range with a slight affinity for the α isoform (*K*_I_ HpCAα = 8.4 μM, *K*_I_ HpCAβ = 13.3 μM), whereas thymol was endowed with a focused and selective inhibition of the bacterial β class (*K*_I_ HpCAβ = 3.4 μM, *K*_I_ MgCA >100 μM). These results were comparable to those obtained with CAIs, such as selenazoles [34], and some sulphonamide-based compounds [35]. Similarly, famotidine was characterized by a more potent inhibitory profile in the nanomolar range toward both HpCAs, but it lacked selectivity with respect to human isoforms [25]. In regard to the inhibition of other two bacterial β-CAs from *P. gingivalis* and *S. mutans*, both thymol and carvacrol were less potent with respect to HpCAs. These two targets are important for assessing the effects on the major components of the oral microbiome [36].

Collectively, these data corroborated the choice of phenol-based inhibitors to obtain a broader degree of selectivity within this large family of enzymes. To better assess this isoform selectivity, we also tested them against the *Malassezia globosa* β-CA and found that despite the similar structural scaffold, the two natural compounds exerted different inhibitory effects, only carvacrol being active against this isoform in the low micromolar range (*K*_I_ = 6.8 μM).

### 2.2. In Silico Studies

Docking and molecular dynamic (MD) simulations were performed to study the binding mode of carvacrol and thymol in the active site of the α- and β-CA of *Helicobacter pylori* (HpCAα and HpCAβ) and β-CA of *Malassezia globosa* (MgCA). While the 3D coordinates of HpCAα (PDB 4XFW) [37] are available in the protein data bank, the X-ray solved structure of HpCAβ is not. Thus, comparative modelling was used to build a structural model based upon the known 3D coordinates of type I β-CAs from *Synechocystis* sp. (5SWC) [38], i.e., the template 3D structure with the highest percentage of homology (32%) among those available (Figure S1). A homology model previously reported by us was instead used for MgCA [39,40,41]. Of note, neither structural nor modelling studies have been reported to date for HpCAβ inhibitors.

Phenols act as CAIs anchoring to the zinc-bound water/hydroxide ion [42]. This behavior results in a generally weaker inhibition profile than that observed for the zinc-binders (Table S1) [43]. Unexpectedly, the modelling study did not provide outcomes for thymol docked within the active site of hCA I, hCA II, HpCAα, and MgCA. In addition, no poses were found for carvacrol within hCA I and II due to conformational constrains that prevent the optimal complementarity of the ligand to the receptor (Figures S2–S5), which is in line with the *K*_I_ values (>100 μM).

**Figure 1:**
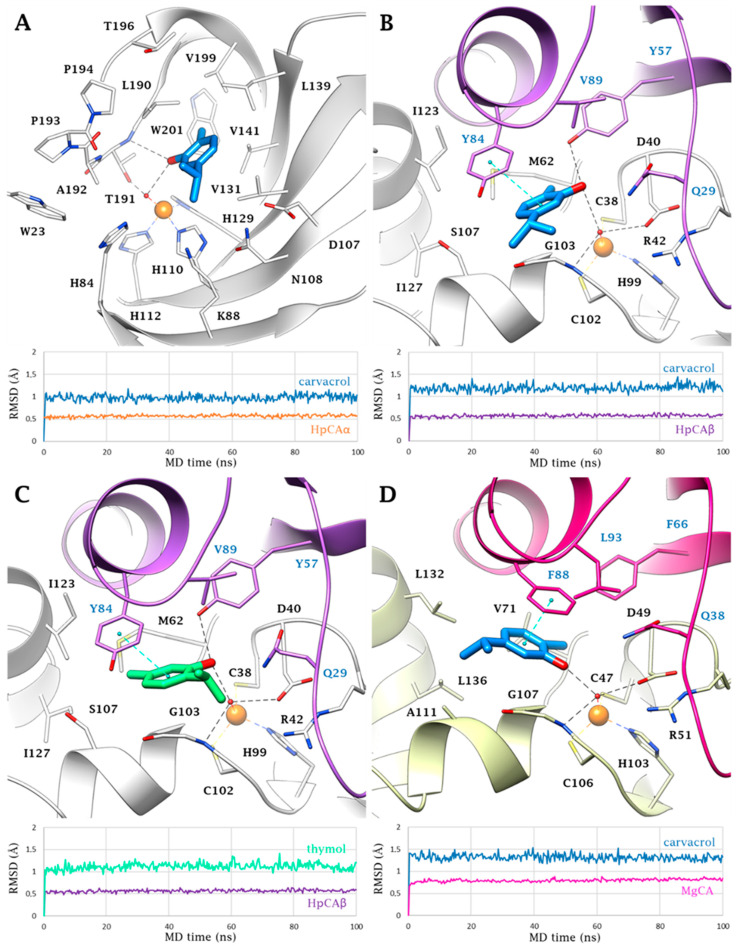
Ligand interactions within (**A**) HpCAα, (**B**,**C**) HpCAβ, and (**D**) MgCA active sites. Carvacrol and thymol are represented in blue and green, respectively. Snapshots from the corresponding MD are shown in each panel together with the trajectories.

Docking solutions for thymol within HpCAβ and carvacrol within HpCAα, HpCAβ, and MgCA (Figures S6–S8) were implemented with three runs of independent 100 ns long MD simulations in order to evaluate the pose stability. In HpCAα, carvacrol is H-bond anchored to the zinc-bound hydroxide ion by the phenol group that, in turn, is in H-bond contact with T191 NH. This interaction network persists for the 81% of the MD time. The ring is lodged in a pocket delimited by hydrophobic residues (V131, V141, L191, and W201) which together maintain the ligand orientation (Figure 1A). In HpCAβ, both ligands can strengthen the anchorage to the zinc-bound OH group thanks to an additional H-bond formed by the OH group and Y57 side chain. MD trajectories confirmed the persistence of the H-bond network either for carvacrol (83%) and thymol (91%) and could provide a reasonable explanation for the 4-fold higher inhibition profile of thymol over carvacrol. In fact, in addition to the more persistent H-bond interactions, thymol stably maintains an extensive network of hydrophobic interactions involving both the 2-isopropyl (Y57, Y84, V89, C102, and G103) and 5-methyl (M62, Y84, and I123; Figure 1C) groups. Although present, the hydrophobic interactions of the 2-methyl (M62 and Y57) and the 5-isopropyl (G103 and S107) groups of carvacrol (Figure 1B) are less persistent and involve fewer residues.

In MgCA, carvacrol is H-bound to the hydroxide Zn-OH group (83% in MD). A π-π stacking occurs between the aromatic rings of the ligand and F88, while π-alkyl contacts exist with V71, F88, and G107 (Figure 1D). The 2-methyl and 5-isopropyl substituents interact with F66 and V71, and with F88, A111, L132, and L136, respectively. It is likely that the size of MgCA over the HpCAβ binding site affects the docking. Good shape complementarity between ligands and MgCA was found only for carvacrol. This could also explain the 2-fold higher inhibition of MgCA over HpCAβ by carvacrol. Instead, internal energy strains prevent the binding of thymol within MgCA.

### 2.3. Determination of the MIC, MBC, and MBIC of Carvacrol, Thymol, and Amoxicillin Versus H. pylori ATCC43504

**Figure 2:**
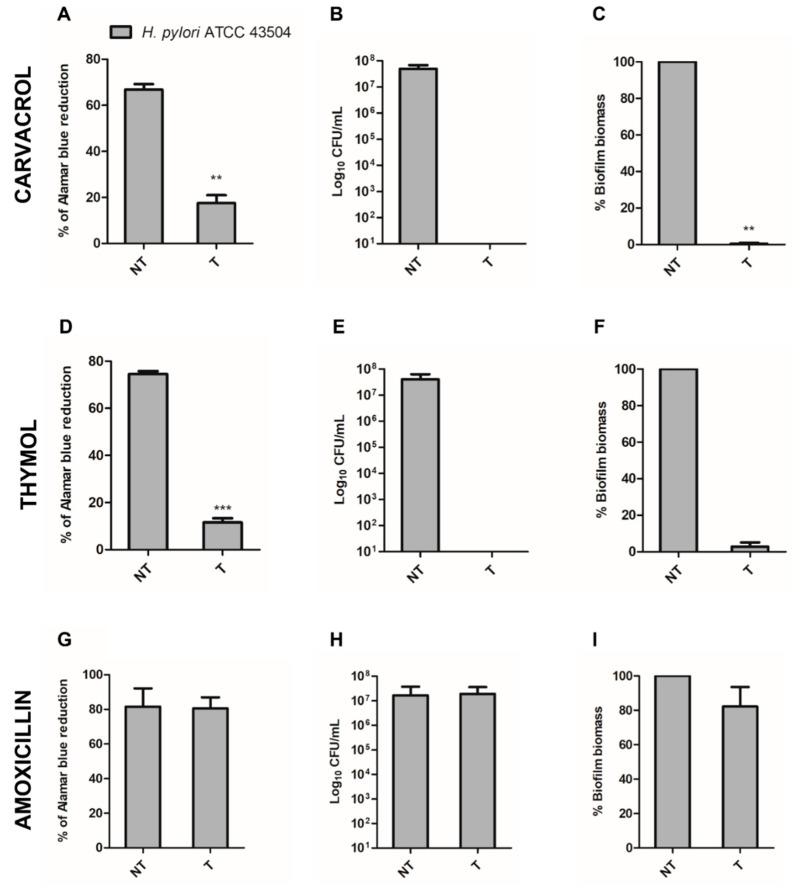
Determination of the MBIC of carvacrol, thymol, and amoxicillin versus *H. pylori* through the alamarBlue assay (**A**,**D**,**G**), the CFU count (**B**,**E**,**H**), and the crystal violet assay (**C**,**F**,**I**). T: treated samples; NT: non-treated samples. ** *p* < 0.005, *** *p* < 0.001 vs. the non-treated sample.

Both carvacrol and thymol, tested versus *H. pylori* ATCC43504, showed an MIC at 128 µg/mL and an MBC at 256 µg/mL (Table 2). These data are comparable to the results previously obtained by Sisto et al., although the authors used a different culture medium [26,27]. To determine the MBIC, sub-MIC concentrations corresponding to 64 µg/mL of both carvacrol and thymol were used. These concentrations, expressed in µg/mL, when converted to µM (about 426 µM for thymol and carvacrol) could provide, after administration, a high inhibitory ability of CA enzymes. In particular, both carvacrol and thymol showed the ability to inhibit the development of the *H. pylori* mature biofilm in respect to the non-treated samples, as demonstrated by the alamarBlue reduction, CFU counts, and crystal violet assay (Figure 2). The percentages of alamarBlue reduction in carvacrol-treated samples and thymol-treated samples were about 80% and 85%, respectively, in respect to the corresponding controls (Figure 2A,D). The inhibition of biofilm development in the treated samples was confirmed by the CFU count. No CFUs were observed in the carvacrol-treated or thymol-treated samples; on the contrary, the untreated samples showed 4–6 × 10^7^ CFU/mL (Figure 2B,E). The treated and the untreated *H. pylori* ATCC43504 samples were then stained with crystal violet to evaluate the presence of biofilm biomass. The biomass percentage of both carvacrol-treated and thymol-treated samples was significantly lower in respect to the untreated samples, confirming the lack of biofilm formation in the presence of sub-MIC concentrations of the two CA inhibitors (Figure 2C,F).

*H. pylori* biofilm was also treated with sub-MIC concentrations of amoxicillin as control. Amoxicillin showed its MIC value at 0.032 µg/mL and MBC value at 0.032 µg/mL, as also previously reported by Grande et al. [44]. At sub-MIC concentrations, the amoxicillin did not inhibit *H. pylori* biofilm formation as shown by the alamarBlue reduction, CFU counts, and crystal violet assay (Figure 2G–I).

**Figure 3:**
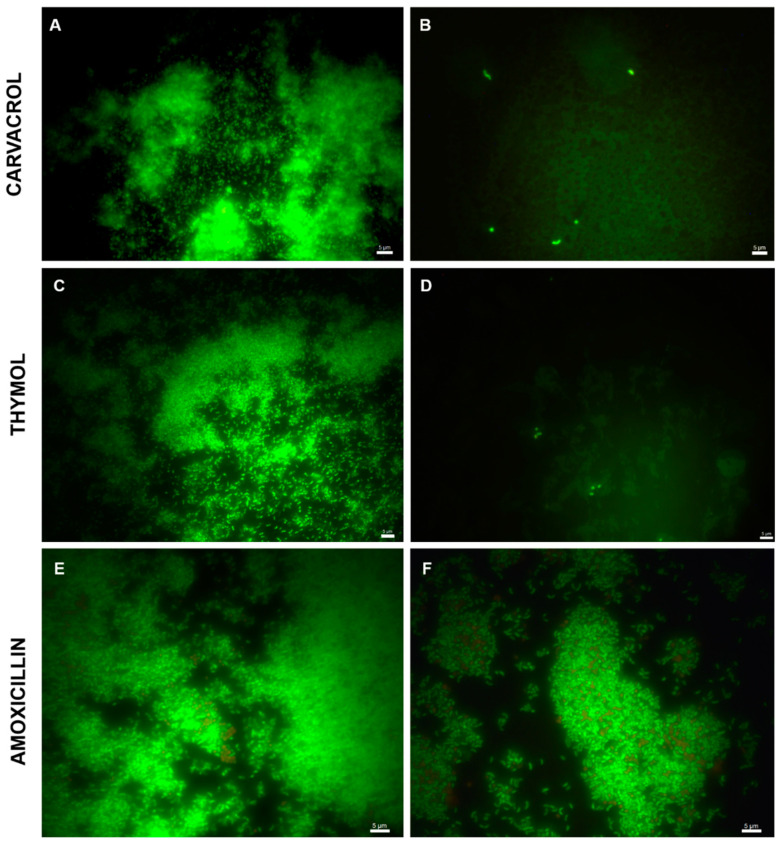
Representative images of *H. pylori* ATCC43629 biofilm after 72 h of incubation. The biofilms have been observed by using a fluorescence microscope. (**A**,**C**,**E**) untreated biofilm and (**B**) carvacrol-treated sample, (**D**) thymol-treated sample, and (**F**) amoxicillin-treated sample. Green fluorescence indicated the presence of live cells, and red fluorescence indicated the presence of dead cells. The presence of dead cells was negligible. Bar: 5 µm.

Fluorescence microscopy analysis of the untreated samples, after 72 h of incubation, showed the development of a mature biofilm characterized by the aggregation of live cells, indicated by a marked green fluorescence, surrounded by an abundant EPS matrix (Figure 3A,C). In contrast, the carvacrol- and thymol-treated samples showed no biofilm formation, with few live cells adhering to the bottom of the plate, most of them with a coccoid morphology (Figure 3B,D). The amoxicillin-treated samples showed a well-structured biofilm with some dead cells (Figure 3F).

### 2.4. Flow Cytometry Detection of Cell Viability

The viability of the cells derived by treated and untreated *H. pylori* samples was also assessed by using flow cytometry. The samples were stained using the Live/Dead™ BacLight™ Bacterial Viability Kit (Life Technologies Carlsbad), based on SYTO 9 and propidium iodide staining for flow cytometry purposes. The untreated samples showed a high percentage of viability corresponding to almost 82% (1.73 × 10^7^ live cells/mL). Flow cytometry analyses confirmed CFU counts corresponding to 4–6 × 10^7^ CFU/mL. Carvacrol-treated samples showed a viability of 35%, corresponding to 3.80 × 10^4^ live cells/mL. These values differed from the values of the CFU count in which no CFUs were detected (Figure 2B) probably because the flow cytometry, different by cultural methods, is also capable of detecting viable but non-culturable (VBNC) cells. The thymol-treated samples showed a viability around 32%, corresponding to 3.00 × 10^5^ live cells/mL. These values differed from the values of the CFU count in which no CFUs were detected for the biofilm phenotype (Figure 2E) for the same reason aforementioned. The amoxicillin-treated samples showed a viability around 64%, corresponding to 3.18 × 10^6^ live cells/mL. These values were comparable to the values of the CFU count in which 1.92 × 10^7^ CFUs were detected for the biofilm phenotype (Figure 2H).

### 2.5. OMVs and OMVs-eDNA^+^ Obtained by the Planktonic and Biofilm Phenotypes of H. pylori ATCC43504 Quantified by Using Flow Cytometry

The detection and quantification of OMVs from planktonic and biofilm phenotypes were performed on the whole sample (cells *plus* vesicles). The flow cytometry method for the detection of OMVs allowed for identifying vesicles with an average size ranging from 100 to 200 nm in all the analysed samples. A large percentage (~30–60%) of OMVs of untreated samples were associated with eDNA both in planktonic and biofilm phenotypes (Table 3).

**Figure 4:**
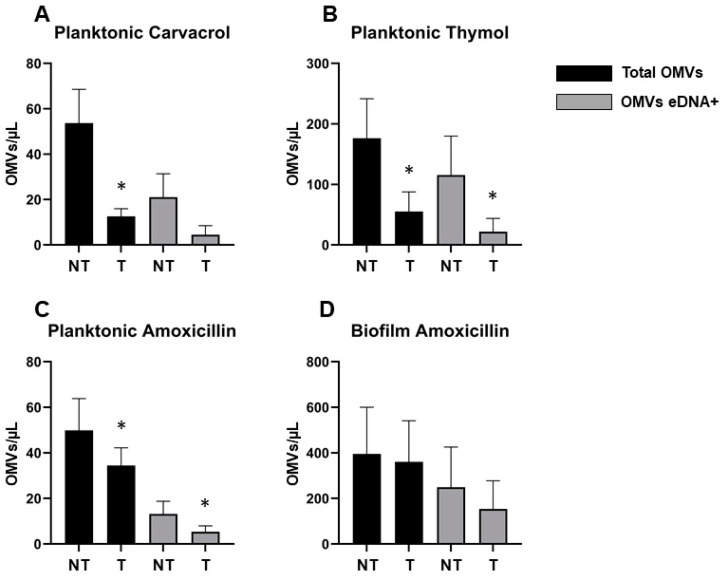
OMV count performed by flow cytometry. The graphs represent the concentration of total and eDNA-containing pOMVs and bOMVs, obtained from treated (T) and untreated (NT) samples with sub-MIC concentrations of carvacrol (**A**), thymol (**B**), and amoxicillin (**C**,**D**). * *p* < 0.05 vs. the non-treated sample.

The total pOMVs detected in the samples treated with carvacrol showed a lower count with respect to the relative control, while no differences were found in pOMV eDNA^+^. The samples treated with sub-MIC concentrations of thymol instead showed a significative difference in total pOMVs and pOMV eDNA^+^ counts. In both cases, extracellular vesicles of biofilm phenotype were not analysed given that no biofilm and no CFUs were detected as confirmed by CV assay, metabolic assay, CFU counts, and fluorescent microscopy analysis (Figure 2 and Figure 3). The samples treated with sub-MIC concentrations of amoxicillin showed a decrease in the production of total pOMVs and pOMV eDNA^+^ with respect to the untreated samples (Figure 4). Instead, no differences in terms of total bOMVs and bOMV eDNA^+^ were detected between amoxicillin-treated and untreated samples (Figure 4 and Supplementary Materials).

To further assess the noxious effect of carvacrol and thymol on the microbiota after oral administration and keeping in mind the mechanism of the antibacterial action based on the carbonic anhydrase inhibition, we tried to explore the presence of this enzyme in the most representative probiotic bacteria often administered to re-stablish eubiosis in the host. Indeed, in simpler organisms, such as bacteria, Archaea, and cyanobacteria, α-, β-, γ-, and ι-CAs are present, with the function of balancing the CO_2_/HCO_3_^−^ concentration ratio and having a role in carbon dioxide fixation. The distribution of the CAs resulted in being quite varied in the probiotic strains reported in Table 4.

For example, the genome of *L. rhamnosus* GG, *L. casei* DG, and *L. acidophilus* encodes only for the α-CA class. In contrast, *B. animalis*, *B. lactis*, and *B. longum* contained only genes for the β-CAs. Intriguingly, *L. reuteri* resulted in the absence of genes encoding for α and β-CAs (Table 4). A common feature of the Gram-negative α-CAs known to date is the presence of an *N*-terminal signal peptide, which suggests a periplasmic or extracellular location and a possible physiological role in CO_2_ uptake processes [45]. Furthermore, the lack of α-CAs in Gram-negative bacteria could be compensated by the presence of β- or γ-CAs characterized by a signal peptide, which may have a periplasmic localization and a role similar to that described for the α-forms [4]. The α- and β-CAs identified in the genome of the probiotics mentioned above are characterized by the absence of the signal peptide. This is not a surprise since Gram-positive bacteria lack an outer membrane.

**Figure 5:**
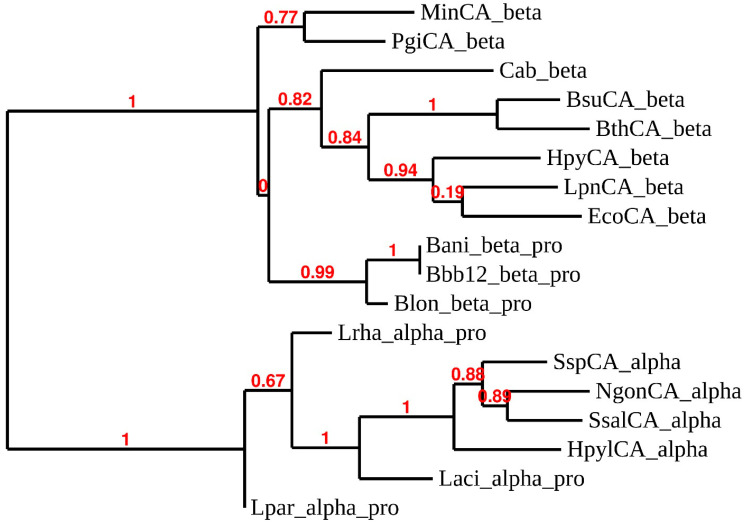
Phylogenetic analysis of CAs from various microorganisms. The dendrogram was obtained using amino acid sequences of probiotic strains in Table 4, and the α-CAs and β-CAs coming from multiple species of bacteria. Bootstrap values of 100 replicates are reported at branch points. Legend: see Table S2 for sequence accession numbers, CA-class, cryptonyms, and organisms considered.

We have constructed a phylogenetic tree to better investigate the evolutionary relationship of α- and β-CAs identified in the probiotic strains considered here. The dendrogram shows that the α-CAs from the probiotic strains seemed closely associated with each other, except that they formed distinct branches (Figure 5) in the α-CAs cluster. The probiotic α-CAs can be considered as transition amino acid sequences from which the other α-CAs from the Gram-negative have originated. This is corroborated by Gupta’s hypothesis, which maintains that Gram-positive bacteria occupy an intermediate position between Archaea and Gram-negative bacteria. This latter group has evolved from Gram-positive bacteria [46]. Similarly, the β-CAs from probiotics seem to have arisen before the other bacterial CAs, except for two β-CAs, MinCA-beta and Pgi_CAbeta.

### 2.6. Carvacrol, Thymol, and Amoxicillin Inhibition on Probiotic Bacteria (Lactobacillus reuteri DSM 17938, Lactobacillus rhamnosus GG ATCC 53103, and Lactobacillus acidophilus ATCC SD5214) Growth

The MIC values of carvacrol, thymol, and amoxicillin versus three probiotic bacteria were determined by the microdilution method. Carvacrol displayed an antibacterial effect against *L. reuteri* DSM 17938 at an MIC corresponding to 8 mg/mL, while thymol had an antibacterial effect at a concentration of 4 mg/mL. Regarding the MIC value of both CA inhibitors versus *L. rhamnosus* GG ATCC 53103 and *L. acidophilus* ATCC SD5214, it was greater than the maximum concentration tested (16 mg/mL). Therefore, the exact value of the MBC of the CA inhibitors versus probiotic bacteria was not determined (Table 2). Contrariwise, the MIC value of amoxicillin was determined at 16 µg/mL for *L. reuteri* DSM 17938, at 0.25 µg/mL for *L. acidophilus* ATCC SD5214, and at 1 µg/mL for *L. rhamnosus* GG ATCC 53103. The MBC value of amoxicillin was at 64 µg/mL for *L. reuteri* DSM 17938, at 0.25 µg/mL for *L. acidophilus* ATCC SD5214, and at 1 µg/mL for *L. rhamnosus* GG ATCC 53103 (Table 2).

## 3. Discussion

The worldwide increase of *H. pylori* strains resistant to the main antimicrobials commonly used in clinical therapy, mainly clarithromycin and amoxicillin, represents a huge concern. The current consensus guidelines recommend the eradication of the microorganism in symptomatic individuals [47,48]. The use of the standard triple therapy consisting of a proton pump inhibitor (PPI) or ranitidine, bismuth citrate, and two antibiotics, chosen among amoxicillin, clarithromycin, and metronidazole, has raised some concerns due to increased eradication failure rates [49]. The treatment failure might be due to several factors such as the antibiotic inactivation because of low stomach pH, the lack of patient compliance, genetic-acquired resistance to the antibiotics, and the capability of *H. pylori* to develop a biofilm [50]. The EPS biofilm matrix protects the bacterial cells by the external stressing stimuli, including the attack from the antimicrobials, which met difficulties in penetrating a biofilm [51]. Tolerance to antimicrobial drugs can be lost when a biofilm is dispersed, and microbial cells return to the planktonic phase. Therefore, the identification of housekeeping gene products which are crucial for the growth and survival of *H. pylori* and the differences of these enzymes with respect to α-CAs found in humans (hCA I, II, and VI) or β-CAs detected in other bacteria (*P. gingivalis*, *S. mutans*) and fungi (*Malassezia globosa*) might represent a useful strategy for the design of new selective inhibitors [44,52]. Furthermore, the periplasmic location of HpαCA makes it a more druggable target, as the inhibitors do not have to cross the cytoplasmic membrane.

So far, few studies have been devoted to finding effective in vitro and in vivo selective CAIs targeting pathogens. Starting from the antimicrobial activity exerted by natural compounds, in the present work, we focused on the anti-*H. pylori* activity of the two most important secondary metabolites in plants. We demonstrated that carvacrol and thymol, two well characterized and selective HpCA inhibitors, are capable of preventing the release of OMVs correlated to an inhibition in biofilm formation as shown by the fluorescent microscopy images. *H. pylori* OMVs are associated with eDNA, preventing the degradation of the nucleic acid and suggesting a possible role in the promotion of the horizontal gene transfer [21,53]. Amoxicillin, one of the most used antibiotics in therapy, did not show any effect in the inhibition of vesicles blebbing in the biofilm phenotype; indeed, in this case, the amoxicillin-treated samples exhibited a similar behavior to the untreated samples. The data obtained demonstrated that a large percentage of the OMVs produced in the two phenotypes were associated with eDNA. The treatment with the two different HpCA inhibitors induced the decrease of total pOMVs. Therefore, CA inhibitors may contribute to limiting the spread of the antibiotic resistance as well as the pathogenicity of the microorganism, since the OMVs carry many virulence factors such as the CagA protein [54]. The results obtained also suggest a new role played by CAs, namely the ability to inhibit the release of eDNA as previously observed in non-tuberculous mycobacteria (NTM) [55]. The authors demonstrated that bicarbonate positively affects eDNA export in NTM; in particular, they investigated the impact on the eDNA export adding the CA inhibitor ethoxzolamide due to the abundant carbonic anhydrase presence in eDNA-containing biofilms of the surface-exposed proteome of *M. avium*.

In the case of *H. pylori,* the eDNA is associated with OMVs and represents a key component since it is involved in “bridging OMV-OMV and OMV-cell interactions”, supporting the stability of the biofilm as previously mentioned [21]. The inhibition of eDNA export stopped the vesiculation and thus biofilm development. The inhibition of *H. pylori* biofilm formation has been demonstrated by using different methods consisting of AlamarBlue metabolic assay, CFU count, CV assay, and fluorescent microscopy analysis, which showed concordant results. Since OMVs are a component of the *H. pylori* biofilm matrix, we assume that the inhibition of the vesiculation process induces a reduction of cell aggregation and adhesion; therefore, the non-adherent cells in the CA-inhibitor-treated samples remain in the planktonic phenotype. On the other hand, the role of OMV-eDNA associated in the cell–cell aggregation has been previously demonstrated [21].

The microbiota is constituted by a community of microorganisms that colonize the same habitat and cooperate with each other, co-existing with potential pathogens; unfortunately, when the established equilibrium fails, infections might develop. The interaction of *H. pylori* with the human microbiota is complex, and the administration of antimicrobial therapies should take into account the effect on the microbiota [44,52]. Thus, it would be desirable to test them for selective toxicity. In the present work, the potential antimicrobial activity of the two HpCA inhibitors versus three probiotic bacteria encoding for CAs has also been evaluated.

Interestingly, the MIC of HpCA inhibitors versus CA-encoding probiotic bacteria was surprisingly high, equal to 4–8 mg/mL for *L. reuteri* DSM 17938 and >16 mg/mL for *L. rhamnosus* GG ATCC 53103 and *L. acidophilus* ATCC SD5214. These results demonstrated a selective toxicity versus the pathogen *H. pylori* (SI of 62.5 and 32.3 for carvacrol and thymol versus *L. reuteri*, respectively, and >125 for both *L. acidophilus* and *L. rhamnosus*), so much so that we can hypothesize a possible association of the CA inhibitors with those probiotic species, obtaining a potential synergistic effect of the two components to implement the effectiveness of a possible anti-*H. pylori* therapy. Amoxicillin displayed an SI of 500 versus *L. reuteri* DSM 17938, 7.81 versus *L. acidophilus* ATCC SD5214, and 31.25 versus *L. rhamnosus* GG ATCC 53103, but in a lower range of clinically useful concentration; thus, it can easily disrupt the microbial balance of the gut, resulting in disease and the development of antimicrobial resistance.

Finally, thymol and carvacrol were shown to display low toxicity against AGS cells (IC_50_ 200 ± 6.5 μM and 300 ± 6.5 μM, respectively) [26,27], thus ensuring a safe administration by the oral route and after contact with the stomach environment. Overall, the data indicated that thymol and carvacrol have a double selectivity over human cells and carbonic anhydrases.

## 4. Methods

### 4.1. Chemicals

Carvacrol (99% purity), thymol (>98.5% purity), and amoxicillin (pharmaceutical primary standard) were obtained from Sigma-Aldrich (Milan, Italy).

### 4.2. Carbonic Anhydrase Inhibition Studies

An applied photophysics stopped-flow instrument was used for assaying the CA catalyzed CO_2_ hydration activity. Phenol red (at a concentration of 0.2 mM) was used as an indicator, working at the absorbance maximum of 557 nm, with 20 mM Hepes (pH 7.4) for α-class as a buffer, 20 mM TRIS (pH 8.3) for β-class as a buffer, and 20 mM Na_2_SO_4_ (for maintaining constant ionic strength), following the initial rates of the CA-catalyzed CO_2_ hydration reaction for a period of 10–100 s. The CO_2_ concentrations ranged from 1.7 to 17 mM for the determination of the kinetic parameters and inhibition constants. The non- catalyzed CO_2_ hydration was not subtracted from these curves and accounts for the remaining observed activity even at high concentration of inhibitor, being in the range of 16–25%. However, the background activity from the uncatalyzed reaction is always subtracted when IC_50_ values are obtained by using the data analysis software for the stopped-flow instrument. Enzyme concentrations ranged between 5 and 10 nM. For each inhibitor, at least six traces of the initial 5–10% of the reaction were used for determining the initial velocity. The uncatalyzed rates were determined in the same manner and subtracted from the total observed rates. Stock solutions of the inhibitor (0.1 mM) were prepared in distilled–deionized water, and dilutions up to 0.01 nM were done thereafter with the assay buffer. Inhibitor and enzyme solutions were preincubated together for 15 min at room temperature prior to the assay to allow for the formation of the E–I complex. The inhibition constants were obtained by non-linear least-squares methods using PRISM 3 and the Cheng–Prusoff equation, as reported earlier, and represent the mean from at least three different determinations. All CA isoforms were recombinant proteins obtained in house, as reported earlier [56,57].

### 4.3. Molecular Modelling

All crystal structures or homology-built models used for computational studies were prepared using the Protein Preparation Wizard tool implemented in the Schrödinger suite, assigning bond orders, adding hydrogens, deleting water molecules, and optimizing H-bonding networks [58]. The energy minimization protocol with a root mean square deviation (RMSD) value of 0.30 Å was applied using an Optimized Potentials for Liquid Simulation (OPLS3e) force field [59,60,61].

SWISS MODEL [62] platform was used to identify the template 3D coordinates (type I β-CA from *Synechocystis* sp.) with the highest percentage of homology. The best scored HM model was built with Prime (v. 5.5) and used in the in silico protocol (the sequence alignment is reported in Figure S1) [39,40].

The 3D ligand structures were prepared by Maestro (v. 11.9) and evaluated for their ionization states at pH 7.4 ± 0.5 with Epik (v. 4.7). The conjugate gradient method in Macromodel (v. 12.3) was used for energy minimization (maximum iteration number: 2500; convergence criterion: 0.05 Kcal/mol/Å^2^). Grids for docking were centered in the centroid of the complexed ligand, and for molecular docking studies, the software Glide SP (v. 8.2; default settings) was used.

Molecular dynamics (MD) simulations were performed using Desmond Molecular Dynamics System (v. 5.7) and OPL3e force field. All systems were solvated in an orthorhombic box using simple point charge water molecules extended 15 Å away from any protein atom around the transmembrane domain helices. The system was neutralized with 0.15 M Cl^−^ and Na^+^ ions. The simulation protocol included a starting relaxation step followed by a final production phase of 50 ns. In particular, the relaxation step comprised the following: (a) a stage of 100 ps at 10 K retaining the harmonic restraints on the solute heavy atoms (force constant of 50 Kcal/mol/Å^2^) using the NPT ensemble with Brownian dynamics; (b) a stage of 12 ps at 10 K with harmonic restraints on the solute heavy atoms (force constant of 50 Kcal/mol/Å^2^), using the NVT ensemble and Berendsen thermostat; (c) a stage of 12 ps at 10 K and 1 atm, retaining the harmonic restraints and using the NPT ensemble and Berendsen thermostat and barostat; (f) a stage of 12 ps at 300 K and 1 atm, retaining the harmonic restraints and using the NPT ensemble and Berendsen thermostat and barostat; (g) a final 24 ps stage at 300 K and 1 atm without harmonic restraints, using the NPT Berendsen thermostat and barostat. The final production phase of MD was run using a canonical NPT Berendsen ensemble at 300 K. During the MD simulation, a time step of 2 fs was used while constraining the bond lengths of H atoms with the M-SHAKE algorithm. The atomic coordinates of the system were saved every 100 ps along the MD trajectory. Protein RMSD, ligand RMSD/RMSF (root mean square fluctuation), ligand torsions evolution, and occurrence of intermolecular H-bonds and hydrophobic contacts were provided by the simulation interaction diagram (SID) implemented in Maestro along with the production phase of the MD simulation. The tool reads the MD trajectory file and identifies ligand/target interactions repeatedly occurring during the simulation time (for instance, a 60% value suggests that the interaction is maintained for the 60% of the MD). Figures were generated with Chimera [63].

### 4.4. Bacterial Strains and Media

*H. pylori* ATCC43504/NCTC11637 reference strain, isolated from the gastric antrum and characterized as cagA^+^ and vacA^+^, was used in the study. It is resistant to metronidazole (MIC range: 64–256 μg/mL), but mostly sensitive to amoxicillin (MIC range: 0.015/0.12 μg/mL), and also to clarithromycin (MIC range: 0.016/0.25 μg/mL), tetracycline (MIC range: 0.125/1 μg/mL), and levofloxacin (MIC range: 0.064 /0.5 μg/mL).

The stock was stored at −80 °C before being thawed at room temperature, plated on Columbia agar base (CA; Oxoid Ltd., Hampshire, UK) supplemented with 10% horse serum (Sigma Aldrich, St. Louis, MO, USA) and 0.25% bacto yeast extract (Oxoid Ltd., Hampshire, UK) and finally incubated at 37 °C for 72 h in a microaerophilic atmosphere (Campy Pak Jar; Oxoid Ltd., Hampshire, UK) [47]. *Lactobacillus reuteri* DSM 17938, kindly provided by BioGaia AB (Stockholm, Sweden), was used in the study and grown as previously indicated [64]. After incubation, the broth culture was diluted in MRSB (DeMan-Rogosa-Sharpe broth) to an optical density (OD_600_) of 0.10, corresponding to approximately 10^7^ Colony Forming Units/mL (CFU/mL). The broth culture was then diluted 1:10 in MRSB and used for the evaluation of the minimum inhibitory concentration (MIC) in the 96-well plate. Each well contained the bacteria at a final concentration of 2–8 × 10^5^ CFU/mL.

### 4.5. Antimicrobial Activity of Carvacrol, Thymol, and Amoxicillin Versus H. pylori ATCC43504

*H. pylori* colonies were collected and inoculated in Brucella Broth (BB; Oxoid Ltd., Hampshire, UK) added with 2% (*v*/*v*) of foetal bovine serum (FBS; Sigma Aldrich, Milan, Italy). The broth cultures were subsequently incubated overnight (ON) at 37 °C under microaerophilic conditions and stirred at 125 rpm (Campy Pak Jar; Oxoid Ltd., Hampshire, UK). After 18 h of incubation, the broth cultures were diluted till OD_600_ of 0.10, corresponding to 2.84 × 10^7^ CFU/mL and further diluted to obtain 5 × 10^5^ CFU/mL per condition. The MIC was determined by the broth micro-dilution method, following the guidelines of the Clinical and Laboratory Standards Institute [65]. Amoxicillin was resuspended in DPBS, diluted in liquid culture medium consisting of BB supplemented with 2% (*v*/*v*) of FBS, and was tested in the range of 0.002–0.128 µg/mL. Carvacrol and thymol were resuspended in dimethyl sulfoxide (DMSO, Sigma-Aldrich, Milan, Italy) and were diluted in the liquid culture medium described above. Serial dilutions of the two HpCA inhibitors were carried out to obtain the concentration range of 16–512 µg/mL, with a residual percentage of DMSO < 0.06%. The controls consisted of: (I) *H. pylori* broth culture; (II) BB plus 2% (*v*/*v*) of FBS, with the addition of the two CA inhibitors or amoxicillin at different concentrations; (III) just BB plus 2% (*v*/*v*) of FBS. Three independent experiments were performed in triplicate. The plates were then incubated at 37 °C for 72 h under microaerophilic conditions. After 72 h incubation, the MIC was determined by the alamarBlue assay (AB; Thermo Fisher Scientific, Waltham, MA, USA), since the lack of turbidity of *H. pylori* broth cultures prevents the eye measurement of the MIC. AlamarBlue was added at 10% in each well, following the manufacturer instructions, and the plates were placed in an incubator for 4 h at 37 °C in microaerophilic conditions. After the incubation, the percentage of alamarBlue reduction of treated and untreated samples was determined, as previously described [66].

The minimum bactericidal concentration (MBC) was determined by using the same wells stained with alamarBlue. Briefly, 100 μL of the bacterial suspensions, taken from the blue-violet wells, were seeded on the selected agar plates and incubated at 37 °C for 3–5 days in microaerophilic conditions. MBC was defined as the lowest concentration of carvacrol or thymol capable of killing the 99.9% of the starting bacterial inoculum.

### 4.6. Antibiofilm Activity of Carvacrol, Thymol, and Amoxicillin Versus H. pylori ATCC43504

The antibiofilm activity was determined by treating *H. pylori*-forming biofilm with sub-MIC concentrations of carvacrol, thymol, and amoxicillin. The two selective CA inhibitors were resuspended in DMSO, while the amoxicillin was resuspended in DPBS. The CA inhibitors and amoxicillin were diluted in liquid culture medium consisting of BB supplemented with 2% (*v*/*v*) of FBS and 0.3% (*w*/*v*) of glucose. The dilution of the CA inhibitors was performed till a concentration of 64 µg/mL, corresponding to the sub-MIC dose, designated as Treated (T), while the amoxicillin was diluted till a sub-MIC concentration of 0.016 µg/mL. For each condition, *H. pylori* was inoculated at a concentration corresponding to approximately 2–8 × 10^5^ CFU per well. The controls consisted of: (I) *H. pylori* grown in BB plus 2% (*v/v*) of FBS and 0.3% (*w*/*v*) of glucose, (II) the two CA inhibitors and amoxicillin at the sub-MIC concentration in BB plus 2% (*v/v*) of FBS and 0.3% (*w*/*v*) of glucose, and (III) just BB plus 2% (*v/v*) of FBS and 0.3% (*w*/*v*) of glucose. The antibiofilm assay was performed in 96-well flat bottom microtiter plates (Eppendorf, Hamburg, Germany). The biofilm formation in the controls and treated samples was evaluated in 35 mm Petri dishes (Eppendorf, Hamburg, Germany) stained with Live/Dead staining and visualized by fluorescence microscope. Three independent experiments were performed in triplicate. The plates were incubated at 37 °C for 72 h under microaerophilic conditions. After 72 h of incubation, the minimum biofilm inhibitory concentration (MBIC) was determined through the alamarBlue assay, the CFU count, and the crystal violet (CV) assay (Sigma Aldrich, St. Louis, MO, USA). In detail, at the end of the incubation, the non-adherent cells, corresponding to the planktonic supernatant, were removed, and the biofilms were washed with 100 µL of DPBS (Sigma Aldrich, St. Louis, MO, USA). AlamarBlue was diluted to 10% in the selected culture medium, and 100 µL was added to each sample in the wells. The samples were incubated for 4 h at 37 °C in microaerophilic conditions, and subsequently the percentage of alamarBlue reduction was determined. The CFU count was carried out starting from the wells stained with alamarBlue. In particular, the biofilm cells were removed by using a cell scraper and resuspended in 1 mL of DPBS corresponding to the stock solution. Serial dilutions of the stock were performed in DPBS, plated on the selected agar, and incubated at 37 °C under microaerophilic conditions for 3–5 days.

The antibiofilm activity was also determined through crystal violet staining following the procedure indicated [67]. Then, the absorbance at 590 nm was measured, and the percentage of biofilm biomass, normalized to the control, was determined. Finally, the antibiofilm activity was demonstrated with Live/Dead staining followed by fluorescence microscope analysis. The biofilms developed in 35 mm Petri dishes were stained with Live/Dead™ BacLight™ Bacterial Viability Kit (Life Technologies Carlsbad, CA, USA) as previously reported [68] and immediately visualized at the fluorescence microscope.

### 4.7. Flow Cytometry Analysis of Cell Viability

Planktonic cells derived by carbonic anhydrase inhibitor (CAI)-treated and non-treated samples were collected by centrifugation at 10,621× *g* for 10 min and stained using the Live/Dead™ BacLight™ Bacterial Viability Kit (Life Technologies Carlsbad) following the manufacturer’s instructions. In the same way, treated and non-treated biofilms were washed with DPBS, and the cell compartment was removed by cracking, collected by centrifugation, and stained using the Live/Dead™ BacLight™ kit as already mentioned [68]. Samples were immediately acquired by flow cytometry (FACSVerse, Becton Dickinson Biosciences, San Jose, CA, USA), as already described [69].

### 4.8. Flow Cytometry Evaluation of OMVs and OMV eDNA^+^ of the Planktonic and Biofilm Phenotypes

The samples of the planktonic and the biofilm phenotypes in which cell colonies were detected were collected from the CAI/amoxicillin-treated and untreated wells and digested with 1 µL DNaseI for fifteen minutes at room temperature. OMVs were stained with LCD, PHK26, and PicoGreen and analysed by flow cytometry following the procedure described by Puca et al. [68].

### 4.9. Determination of MIC and MBC of Carvacrol, Thymol, and Amoxicillin Versus Probiotic Bacteria (Lactobacillus reuteri DSM 17938, Lactobacillus rhamnosus GG ATCC 53103, and Lactobacillus acidophilus ATCC SD5214)

The MIC and MBC of carvacrol and thymol versus probiotic bacteria were determined in MRSB by using the broth microdilution method in 96-well polystyrene microtiter plates (Eppendorf, Hamburg, Germany) according to the guidelines of the CLSI [65]. Amoxicillin was resuspended in DPBS, diluted in MRSB, and tested in the range of 0.0019–64 µg/mL. carvacrol and thymol were resuspended in DMSO and diluted in MRSB. Serial dilutions of the two CA inhibitors were carried out to obtain the concentration range of 0.125–16 mg/mL, with a residual percentage of DMSO <2%. The controls consisted of: (I) probiotic bacteria grown in MRSB; (II) probiotic bacteria grown in MRSB plus 1.87% DMSO; (III) MRSB with the addition of the two CA inhibitors or amoxicillin at different concentrations; (VI) MRSB plus 1.87% DMSO; (V) only MRSB. Two independent experiments were performed in triplicate. The plates were then incubated at 37 °C for 24 h under anaerobic conditions. The MBC was defined as the lowest concentration of carvacrol, thymol, or amoxicillin that induced a reduction of the initial inoculum ≥99.9% on agar plates and was determined by spreading on MRSA (DeMan-Rogosa-Sharpe agar) 100 µL of sample taken by the wells corresponding to the MIC values. SI values were expressed as MIC probiotic bacteria/MIC *H. pylori* ratio.

### 4.10. Expression and Purification of the CAs

*Escherichia coli* (DE3)-competent cells were transformed with the expression vector containing the CA of interest. Transformed cells were grown at 37 °C and induced with 1 mM isopropyl β-D-1-thiogalactopyranoside (IPTG). ZnSO_4_ was added after 30 min, and after additional growth for 3 h, cells were harvested and disrupted by sonication at 4 °C in 20 mM buffer phosphate, pH 8.0. Following sonication, the sample was centrifuged at 1200× *g* at 4 °C for 30 min. The supernatant was dialyzed against 0.02 M phosphate buffer (pH 8.0) containing 0.01 M imidazole and loaded onto a His-select HF Nickel affinity column (GE Healthcare, Chicago, IL, USA). The column was equilibrated with 0.02 M phosphate buffer (pH 8.0) containing 0.01 M imidazole and 0.5 M KCl at a flow rate of 1.0 mL/min. The recombinant enzyme was eluted from the column with 0.02 M phosphate buffer (pH 8.0) containing 0.5 M KCl and 0.3 M imidazole at a flow rate of 1.0 mL/min. Fractions (0.5 mL) were collected and combined to a total volume of 2.5 mL. Subsequently, the enzyme was dialyzed, concentrated, and analyzed by SDS-PAGE. At this stage of purification, the enzyme of interest was at least 95% pure.

### 4.11. Sequence and Phylogenetic Analysis

Multialignment of amino acid sequences was performed using the program MUSCLE 3.1 (MUltiple Sequence Comparison by Log-Expectation), a new computer program for creating multiple alignments of protein sequences [70]. The dendrogram was constructed using the program PhyML 3.0, searching for the tree with the highest probability [71].

## 5. Conclusions

Recent studies have shown that selected CAIs hamper the growth of *H. pylori* in vitro and in vivo. Thus, these agents might be used as new pharmacologic tools in the management of drug-resistant strains. Carvacrol and thymol are much more isoform-selective CAIs compared to acetazolamide as demonstrated by in vitro and in silico data. Moreover, they effectively inhibit the bacterial growth, the biofilm production at sub-MIC concentrations, the release of OMVs, and the content of associated eDNA, and these results are better than those registered for amoxicillin, allowing us to propose them as new leads for the design of anti-infectives with an innovative mechanism of action. Lastly, the possibility to not interfere at higher concentrations with the presence and survival of three probiotic bacteria opens new scenarios in their selective toxicity and antibiotic-induced gut dysbiosis. Further studies should be performed to better understand the mechanism responsible for inhibiting the release of associated OMV-eDNA as well as the genes and/or proteins involved.

## Figures and Tables

**Table 1:** Inhibition data of hCA I, hCA II, hCA VI, HpCAα, HpCAβ, PgiCAβ, SmuCA, and MgCA with the two natural compounds (carvacrol and thymol) and the standard sulphonamide inhibitor acetazolamide (AAZ) by a stopped-flow CO_2_ hydrase assay.

Compound	Structure	*K*_i_ (μM) ^a^							
		hCA I	hCA II	hCA VI	HpCAα	HpCAβ	PgiCAβ	SmuCA	MgCA
carvacrol	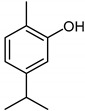	˃100	˃100	˃100	8.4	13.3	28.0	86.6	6.8
thymol	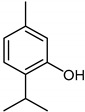	˃100	˃100	˃100	˃100	3.4	48.9	84.9	˃100
AAZ		0.25	0.012	0.011	0.021	0.040	0.214	0.344	40.0

^a^ Mean from three different assays by using a stopped-flow technique, and errors are in the range of ±5–10% of the reported values.

**Table 2:** Determination of the MIC, MBC, and SI * of carvacrol, thymol, and amoxicillin versus *H. pylori* ATCC43504, *L. acidophilus* ATCC SD5214, *L. rhamnosus* GG ATCC 53103, and *L. reuteri* DSM 17938.

Bacteria	MIC (µg/mL)			SI			MBC (µg/mL)		
	Carvacrol	Thymol	Amoxicillin	Carvacrol	Thymol	Amoxicillin	Carvacrol	Thymol	Amoxicillin
*H. pylori*	128	128	0.032				256	256	0.032
*L. acidophilus*	>16,000	>16,000	0.25	>125	>125	7.81	>16,000	>16,000	0.25
*L. rhamnosus*	>16,000	>16,000	1	>125	>125	31.25	>16,000	>16,000	1
*L. reuteri*	8000	4000	16	62.5	31.25	500	>8000	>4000	64

* SI values were expressed as MIC probiotic bacterium/MIC *H. pylori* ratio.

**Table 3:** Percentages of OMV eDNA^+^, on the total OMVs, obtained from the planktonic and biofilm phenotypes, treated (T) and untreated (NT) with sub-MIC concentrations of carvacrol, thymol, and amoxicillin. Percentages were not analysed when OMV-generating cell colonies were not detected in treated samples (-).

Sample	% OMV eDNA^+^/Total OMVs		
	Carvacrol	Thymol	Amoxicillin
Planktonic NT	37.67	62.64	29.79
Planktonic T	32.53	35.47	16.52
Biofilm NT	-	-	55.39
Biofilm T	-	-	36.09

**Table 4:** CA classes and their accession numbers identified in the genome of the considered probiotics.

Probiotic (Gram-Positive)	CA Class		
	α	β	γ
*Lacticaseibacillus paracasei*	WP_003573881.1	-	-
*Lactobacillus reuteri (Limosilactobacillus reuteri)*	-	-	WP_163622737.1
*Lacticaseibacillus rhamnosus*	WP_005704914.1	-	-
*Lactobacillus acidophilus (Lactobacillus plantarum)*	VDH11415.1	-	-
*Bifidobacterium animalis* subsp. *lactis* BLC1	-	AEN75835.1	-
*Bifidobacterium animalis* subsp. *lactis* BB-12	-	ADC85071.1	-
*Bifidobacterium longum* W11	-	WP_071475326.1	-

-: not present in the genome.

## Data Availability

The datasets generated and analysed in the current study are available from the corresponding author on reasonable request.

## Supplementary Materials

The Supplementary Material for this article can be found online at: https://www.mdpi.com/article/10.3390/ijms222111583/s1.
