# Supplementary material for: Selective Inhibition of Helicobacter pylori Carbonic Anhydrases by Carvacrol and Thymol Could Impair Biofilm Production and the Release of Outer Membrane Vesicles
Source: Int J Mol Sci. 2021 Oct 27;22(21):11583. doi: 10.3390/ijms222111583 (PMC8584244; doi:10.3390/ijms222111583)
Supplement: Supplementary file 1 [file ijms-22-11583-s001.zip › ijms-1391980-supplementary.pdf]

Supporting Information

Selective inhibition of *Helicobacter pylori* carbonic anhydrases by carvacrol and thymol could impair biofilm production and the release of Outer Membrane Vesicles

Rossella Grande [1], Simone Carradori [1,*], Valentina Puca [1], Irene Vitale [1], Andrea Angeli [2], Alessio Nocentini [3], Alessandro Bonardi [3], Paola Gratteri [3], Paola Lanuti [4,5], Giuseppina Bologna [4,5], Pasquale Simeone [4,5], Clemente Capasso [6], Viviana De Luca [6] and Claudiu T. Supuran [2]

1 Department of Pharmacy, "G. d'Annunzio" University of Chieti-Pescara, 66100 Chieti, Italy; rossel-la.grande@unich.it (R.G.); valentina.puca@unich.it (V.P.); irene.vitale@unich.it (I.V.)
2 Section of Pharmaceutical and Nutraceutical Sciences, Neurofarba Department, University of Florence, Via U. Schiff 6, 50019 Sesto Fiorentino (Florence), Italy; andrea.angeli@unifi.it (A.A.); claudiu.supuran@unifi.it (C.T.S.)
3 Laboratory of Molecular Modeling Cheminformatics & QSAR, Section of Pharmaceutical and Nutraceutical Sciences, Neurofarba Department, University of Florence, Via U. Schiff 6, 50019 Sesto Fiorentino (Florence), Italy; alessio.nocentini@unifi.it (A.N.); alessandro.bonardi@unifi.it (A.B.); paola.gratteri@unifi.it (P.G.)
4 Department of Medicine and Aging Science, "G. d'Annunzio" University of Chieti-Pescara, 66100 Chieti, Italy; paola.lanuti@unich.it (P.L.); giuseppina.bologna@hotmail.it (G.B.); pasquale.simeone@unich.it (P.S.)
5 Center for Advanced Studies and Technology (CAST), University "G. d'Annunzio" of Chieti-Pescara, 66100 Chieti, Italy
6 Department of Biology, Agriculture and Food Sciences, National Research Council (CNR), Institute of Biosci-ences and Bioresources, 80131 Naples, Italy; clemente.capasso@ibbr.cnr.it (C.C.); viviana.deluca@ibbr.cnr.it (V.D.L.)
* Correspondence: simone.carradori@unich.it

```
                    10         20         30         40         50         60
           ....|....| ....|....| ....|....| ....|....| ....|....| ....|....|
HpCAβ_A  11  ---------- MKAFLGALEF QENEYEELKE LYESLKTKQK PHTLFISCVD SRVVPNLITG
HpCAβ_B  11  ---------- MKAFLGALEF QENEYEELKE LYESLKTKQK PHTLFISCVD SRVVPNLITG
5SWC     1   SGLVPRGSHM QRLIEGLQKF REGYFSSHRD LFEQLSHGQH PRILFICCSD SRVDPNLITQ

                    70         80         90        100        110        120
           ....|....| ....|....| ....|....| ....|....| ....|....| ....|....|
HpCAβ_A  61  TKPGELYVIR NMGNVIPPKT SHKESLSTMA SIEYAIVHVG VQNLIICGHS DCGACGSTHL
HpCAβ_B  61  TKPGELYVIR NMGNVIPPKT SHKESLSTMA SIEYAIVHVG VQNLIICGHS DCGACGSTHL
5SWC     61  SEVGDLFVIR NAGNIIPPYG AANG--GEGA AMEYALVALE INQIIVCGHS HCGAMKGLLK

                   130        140        150        160        170        180
           ....|....| ....|....| ....|....| ....|....| ....|....| ....|....|
HpCAβ_A  121 INDGITKAKT PYIADWIQFL EPIK-EELKN HPQFSNHFAK RSWLTERLNV RLQLNNLLSY
HpCAβ_B  121 INDGITKAKT PYIADWIQFL EPIK-EELKN HPQFSNHFAK RSWLTERLNV RLQLNNLLSY
5SWC     119 LNS--LQEKL PLVYDWLKHT EATRRLVLDN YSHLEGEDLI EVAVAE--NI LTQLKNLQTY

                   190        200        210        220        230
           ....|....| ....|....| ....|....| ....|....| ....|....| ..
HpCAβ_A  180 DFIQERVVNN ELKIFGWHYI IETGRIYNYN FESHFFEPIE ETIKQRKSHE NF
HpCAβ_B  180 DFIQERVVNN ELKIFGWHYI IETGRIYNYN FESHFFEPIE ETIKQRKSHE NF
5SWC     175 PAIHSRLHRG DLSLHGWIYR IEEGEVLAYD GVLHDFVAP
```

Figure S1. Sequence alignment of HpCAβ (target) and type I β-CA from *Synechocystis* sp. (pdb 5SWC, template).

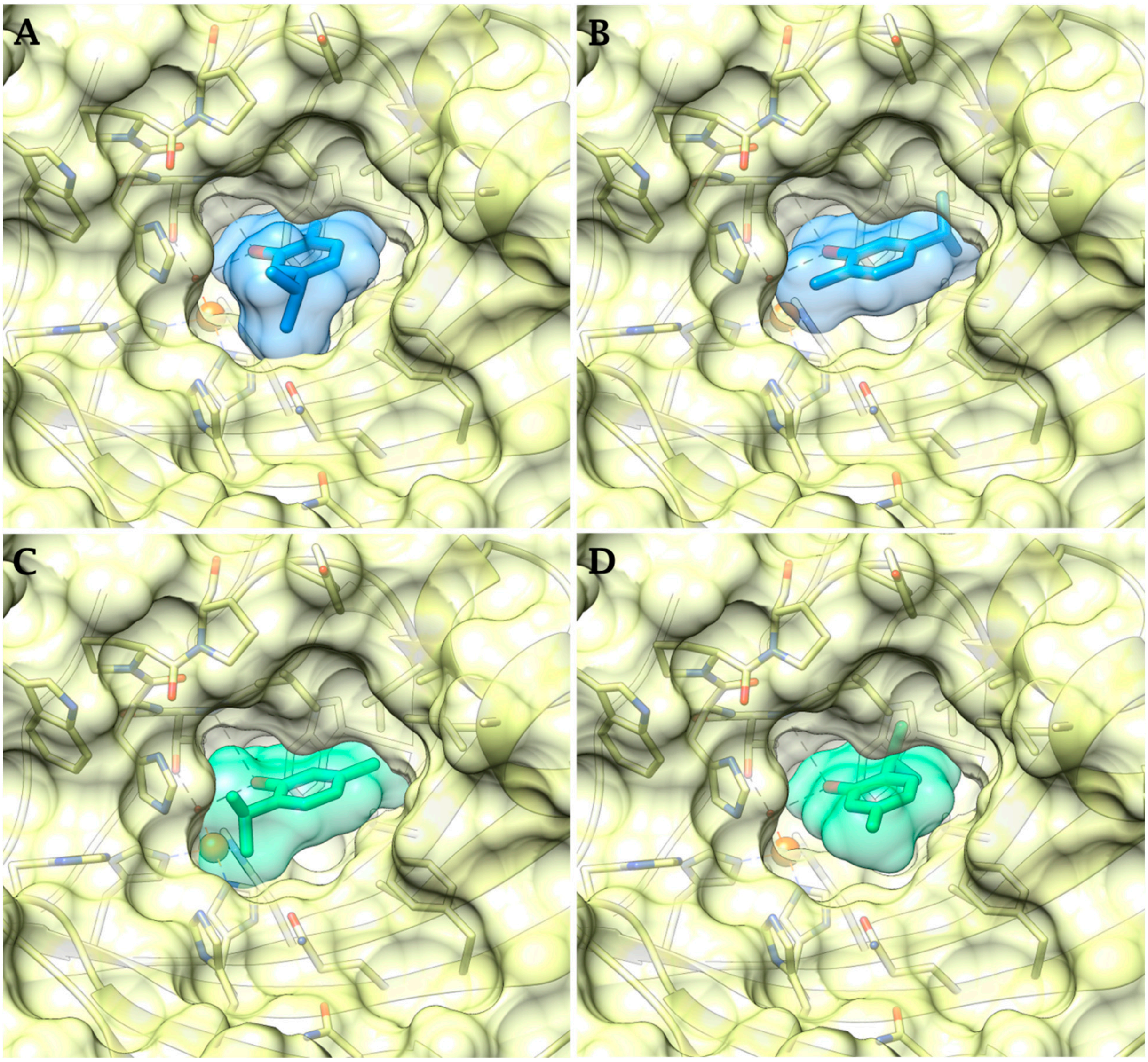

Figure S2. Two possible conformations of (A and B) carvacrol (blue), and (C and D) thymol (green) in hCA I active site, after superimposition with the carvacrol-HpCAα binding mode.

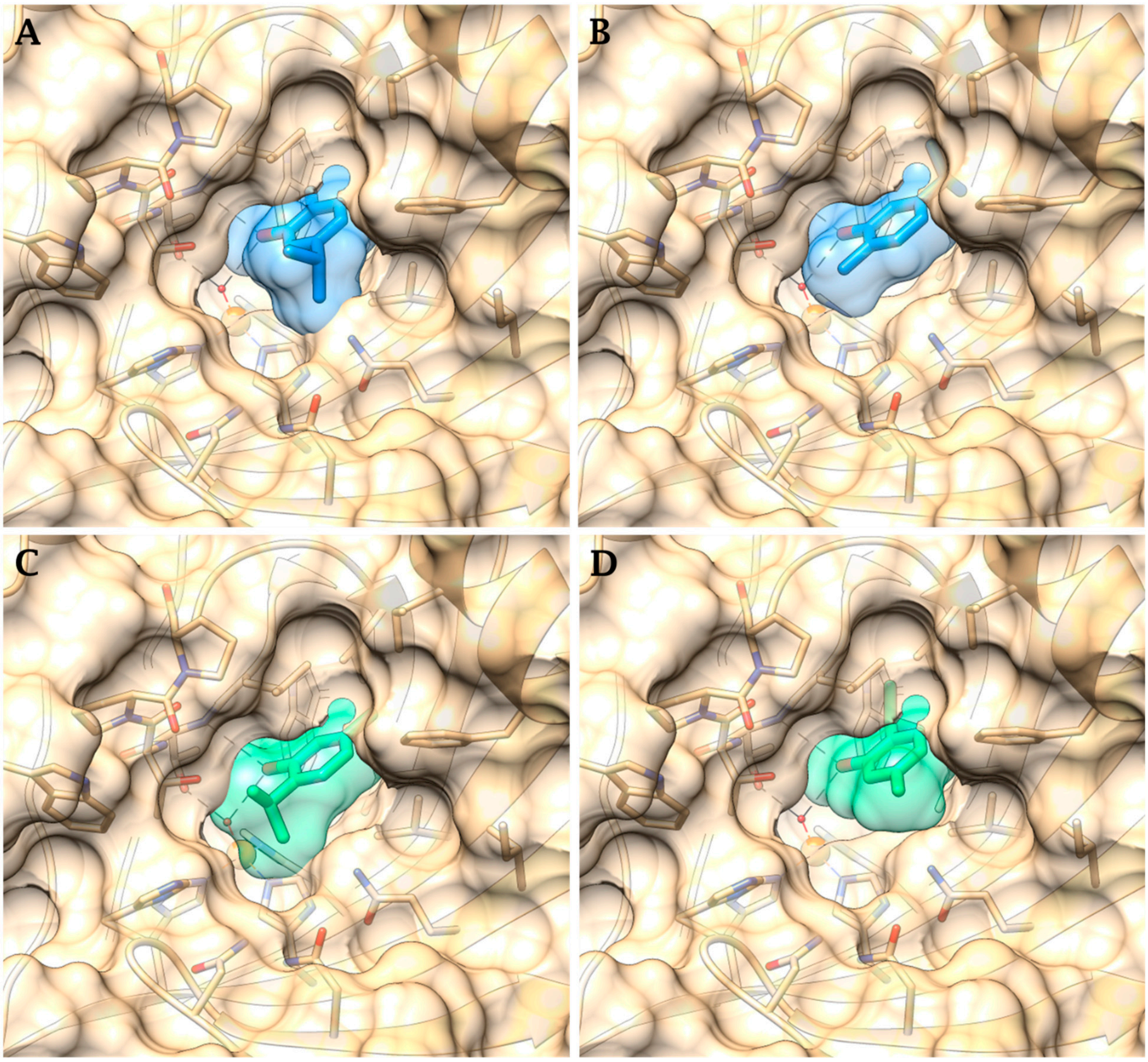

Figure S3. Two possible conformations of (A and B) carvacrol (blue), and (C and D) thymol (green) in hCA II active site, after superimposition with the carvacrol-HpCAα binding mode.

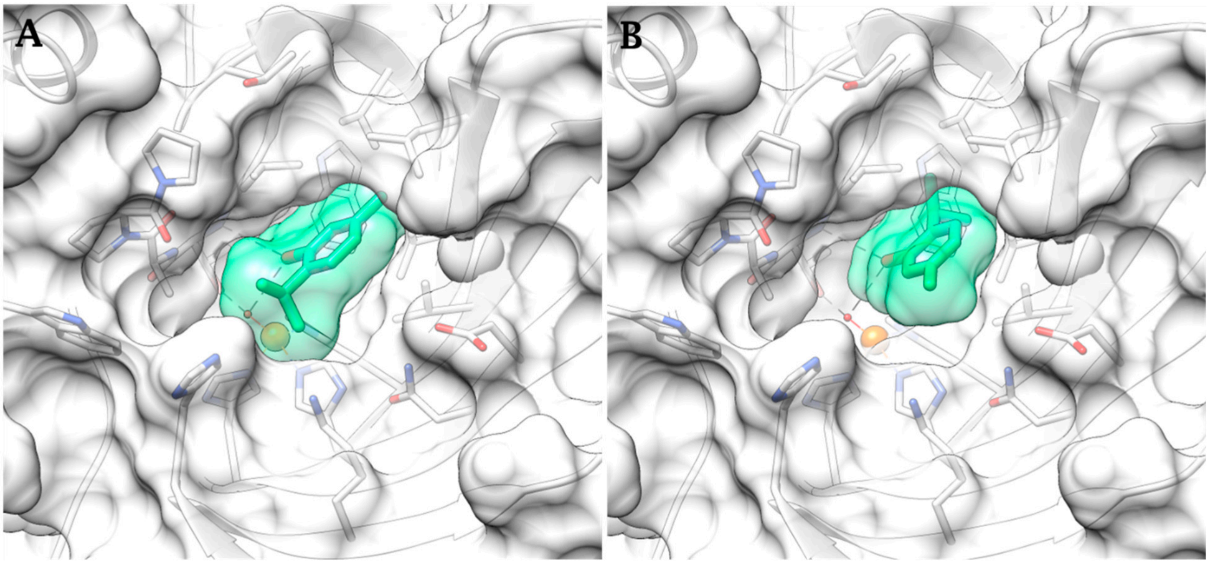

Figure S4. Two possible conformations of (A and B) (green) in HpCAα active site, after superimposition with the carvacrol-HpCAα binding mode.

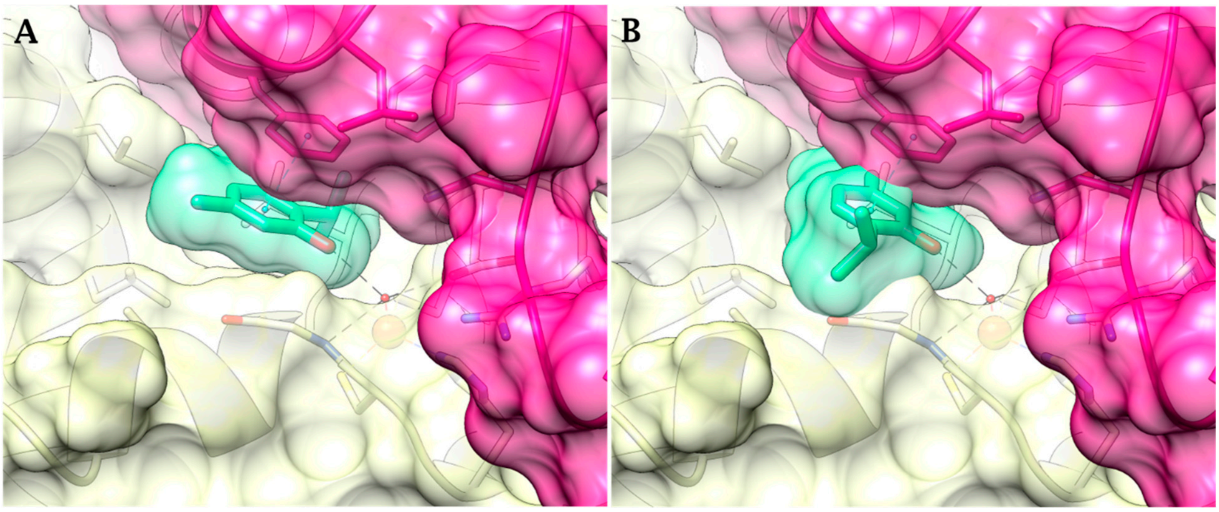

Figure S5. Two possible conformations of (A and B) (green) in MgCA active site, after superimposition with the carvacrol-MgCA binding mode.

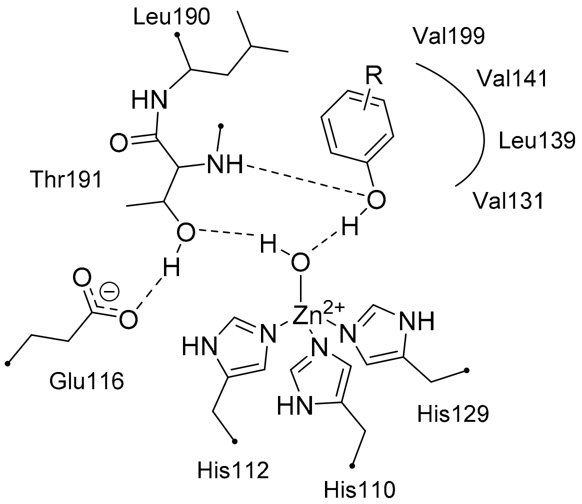

Figure S6. 2D schematic representation of HpCAα residues involved in the interaction with phenols hypothesized by the *in silico* studies.

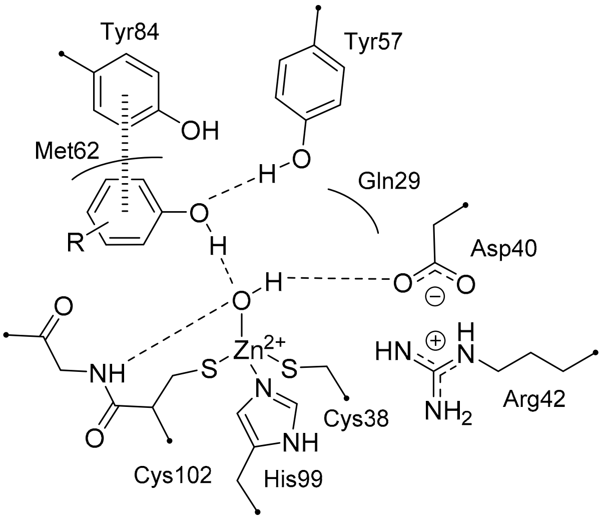

Figure S7. 2D schematic representation of HpCAβ residues involved in the interaction with phenols hypothesized by the *in silico* studies.

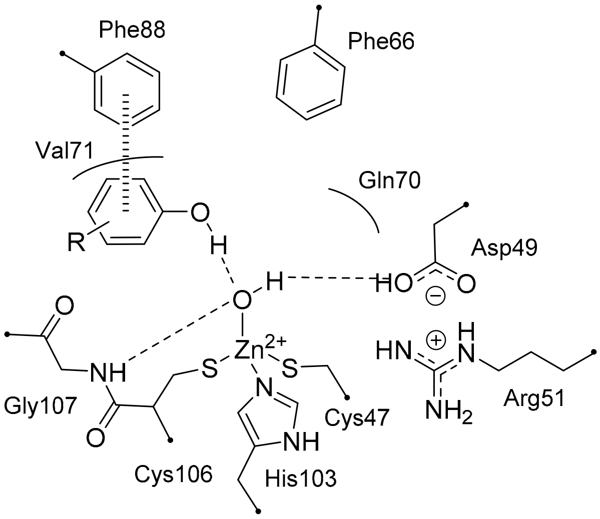

Figure S8. 2D schematic representation of MgCA residues involved in the interaction with phenols hypothesized by the *in silico* studies.

Table S1. Docking and MM-GBSA scores for carvacrol and thymol in hCA I, hCA II, HpCAα, HpCAβ, and MgCA active site.

Target	Ligand	Glide emodel	Docking score	MM-GBSA ΔG bing
hCA I	Carvacrol	–	–	–
	Thymol	–	–	–
hCA II	Carvacrol	–	–	–
	Thymol	–	–	–
HpCAα	Carvacrol	-27.982	-4.727	-24.85
	Thymol	–	–	–
HpCAβ	Carvacrol	-28.491	-5.654	-19.15
	Thymol	-41.021	-7.311	-23.75
MgCA	Carvacrol	-36.839	-6.478	-14.42
	Thymol	–	–	–

Table S2. Organisms, CA classes, acronyms and accession numbers of the amino acid sequences used in the phylogenetic analysis.

Organism	CA class	Acronym	Accession number
Helicobacter pylori	*α*	HpylCA_alpha	WP_010882609.1
Sulfurihydrogenibium sp. YO3AOP1	*α*	SspCA_alpha	WP_012459296.1
Neisseria gonorrhoeae	*α*	NgonCA_alpha	WP_003688976.1
Streptococcus salivarius	*α*	SsalCA_alpha	WP_002888224.1
Lacticaseibacillus paracasei	*α*	Lpar_alpha_pro	WP_003573881.1
Lacticaseibacillus rhamnosus	*α*	Lrha_alpha_pro	WP_005704914.1
Lactobacillus acidophilus (Lactobacillus plantarum)	*α*	Laci_alpha_pro	VDH11415.1
Escherichia coli	*β*	EcoCA_beta	WP_047081292.1
Legionella pneumophila	*β*	LpnCA_beta	WP_011946835.1
Helicobacter pylori	*β*	HpyCA_beta	WP_000642991.1
Burkholderia thailandensis	*β*	BthCA_beta	WP_009893276.1
Brucella suis	*β*	BsuCA_beta	AAN33967.1
Methanothermobacter thermautotrophicus	*β*	Cab_beta	WP_048061095.1
Myroides injenensis	*β*	MinCA_beta	WP_026038855.1
Porphyromonas gingivalis	*β*	PgiCA_beta	WP_012458351.1
Bifidobacterium animalis subsp. *lactis* BLC1	*β*	Bani_beta_pro	AEN75835.1
Bifidobacterium animalis subsp. *lactis* BB-12	*β*	Bbb12_beta_pro	ADC85071.1
Bifidobacterium longum W11	*β*	Blon_beta_pro	WP_071475326.1

Raw data section

Carbonic Anhydrase inhibitory activity:

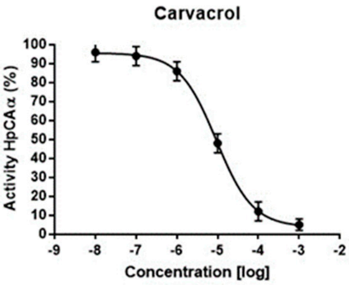

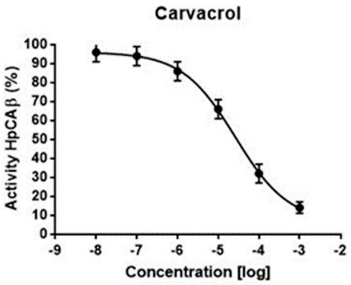

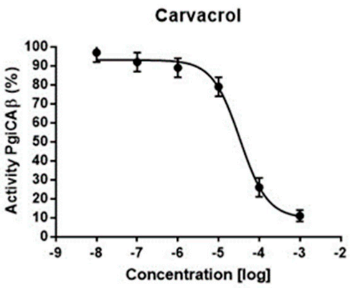

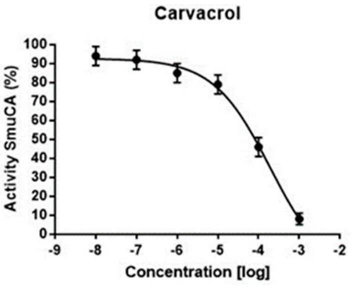

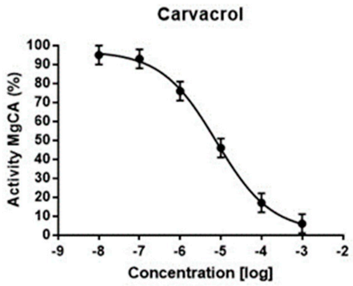

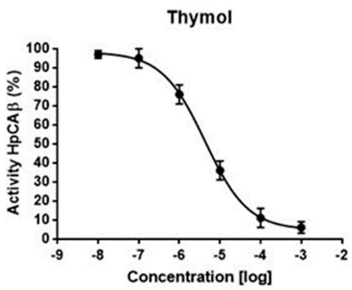

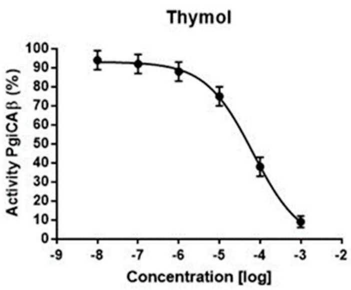

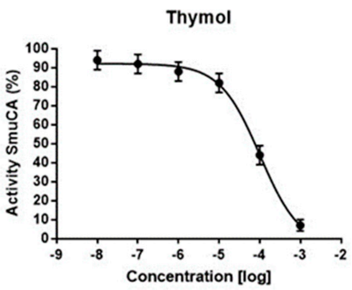

Microbiological assays:

Cristal violet assay for MBIC determination of **amoxicillin** (AMX) *versus Helicobacter pylori*

First experiment

Positive CTRL	AMX Treated	Medium Sterility CTRL	AMX Sterility CTRL
2,0420	2,1027	0,6297	0,6175
1,7917	1,6029	0,4828	0,5998
1,8848	1,1908	0,4802	0,6132
1,4891	1,1937	0,3262	0,6212
1,8742	1,1313	0,5031	0,4522
1,5423	1,2948	0,3912	0,3670
1,7235	1,6220	0,4114	0,3941
1,5109	1,5093	0,6297	0,3863
1,5797	1,3237	0,4828	0,6175
1,6886	1,2467	0,4802	0,5998

	Positive CTRL	AMX Treated	Medium Sterility CTRL	AMX Sterility CTRL
**Mean**	1,7127	1,4218	0,4817	0,5269
**subtraction of whites**	1,2310	0,8949		
	**Positive CTRL**	**AMX Treated**		
**% Biofilm formation**	100	72,70238434		

Second experiment

Positive CTRL	AMX Treated	Medium Sterility CTRL	AMX Sterility CTRL
1,457	1,503	0,675	0,329
1,693	1,075	0,675	0,348
1,164	1,077	0,366	0,348
1,359	1,347	0,318	0,503
1,227	0,901	0,318	0,639
1,145	0,738	0,503	0,524
1,037	1,346	0,634	0,504
1,571	1,200	0,466	0,5998
0,969	1,078	0,675	0,639
0,986	1,020	0,634	0,524

	Positive CTRL	AMX Treated	Medium Sterility CTRL	AMX Sterility CTRL
**Mean**	1,2609	1,1285	0,5265	0,4958
**subtraction of whites**	0,7344	0,6326		

	Positive CTRL	AMX Treated
**% Biofilm formation**	**100**	**86,14613688**

AlamarBlue assay for MBIC determination of **amoxicillin** (AMX) *versus Helicobacter pylori*

First experiment	**Positive CTRL**	**AMX Treated**	**Medium Sterility CTRL**	**AMX Sterility CTRL**
570 nm	**0,5972**	**0,5745**	**0,4365**	**0,4028**
	**0,5977**	**0,6295**	**0,4369**	**0,4074**
	**0,6014**	**0,633**	**0,3937**	**0,4205**
	**0,6108**	**0,6125**	**0,3971**	**0,4432**

	**Positive CTRL**	**AMX Treated**	**Medium Sterility CTRL**	**AMX Sterility CTRL**
600 nm	**0,2101**	**0,1995**	**0,5241**	**0,4968**
	**0,1914**	**0,1859**	**0,5338**	**0,5007**
	**0,2044**	**0,2001**	**0,4867**	**0,5167**
	**0,2066**	**0,1983**	**0,492**	**0,5373**

	**Positive CTRL**	**AMX Treated**	**Medium Sterility CTRL**	**AMX Sterility CTRL**
**(570 nm)**	**0,6018**	**0,6124**	**0,4161**	**0,4185**
**(600 nm)**	**0,203125**	**0,19595**	**0,50915**	**0,512875**

	**Positive CTRL**	**AMX Treated**
**MBIC**	**74,03425314**	**75,95753515**

Second experiment	**Positive CTRL**	**AMX Treated**	**Medium Sterility CTRL**	**AMX Sterility CTRL**
570 nm	**0,688**	**0,644**	**0,42**	**0,426**
	**0,69**	**0,685**	**0,43**	**0,436**
	**0,644**	**0,694**	**0,415**	**0,445**
	**0,681**	**0,7**	**0,387**	**0,434**

	**Positive CTRL**	**AMX Treated**	**Medium Sterility CTRL**	**AMX Sterility CTRL**
600 nm	**0,182**	**0,203**	**0,513**	**0,522**
	**0,171**	**0,166**	**0,528**	**0,535**
	**0,168**	**0,169**	**0,473**	**0,541**

	**0,176**	**0,174**	**0,522**	**0,53**

	**Positive CTRL**	**AMX Treated**	**Medium Sterility CTRL**	**AMX Sterility CTRL**
**(570 nm)**	**0,6758**	**0,6808**	**0,4130**	**0,4353**
**(600 nm)**	**0,17425**	**0,178**	**0,509**	**0,532**

	**Positive CTRL**	**AMX Treated**
**MBIC**	**89,03962295**	**85,6201168**

CFU count for MBIC determination of **amoxicillin** (AMX) *versus Helicobacter pylori*

	**Non treated (CFU/mL)**	**AMX Treated (CFU/mL)**
First experiment	**1,40E+06**	**8,10E+06**
	**3,80E+06**	**6,70E+06**
Second experiment	**1,51E+07**	**1,93E+07**
	**4,60E+07**	**4,25E+07**

Cristal violet assay for MBIC determination of **carvacrol (**CAR**)** *versus Helicobacter pylori*

**First experiment**	**Positive CTRL**	**CAR Treated**	**Medium Sterility CTRL**	**CAR Sterility CTRL**
	**0,6433**	**0,2253**	**0,2343**	**0,2004**
	**0,84**	**0,1881**	**0,2144**	**0,1896**
	**0,9323**	**0,1874**	**0,1857**	**0,1815**
	**0,7468**	**0,198**	**0,2017**	**0,1807**
	**0,7606**	**0,1795**	**0,2162**	**0,2358**
	**0,6639**	**0,1976**	**0,1881**	**0,1846**
	**0,7905**	**0,1969**	**0,1886**	**0,1807**
	**0,7726**	**0,2031**	**0,2192**	**0,2004**
	**0,893**	**0,1961**	**0,1881**	**0,2198**
	**0,7718**	**0,1912**	**0,1886**	**0,1846**

	**Positive CTRL**	**CAR Treated**	**Medium Sterility CTRL**	**CAR Sterility CTRL**
**Mean**	**0,78148**	**0,19632**	**0,20249**	**0,19581**
**subtraction of whites**	**0,57899**	**0,00051**		
	**Positive CTRL**	**CAR Treated**		
**% Biofilm formation**	**100**	**0,088084423**		

**Second experiment**	**Positive CTRL**	**CAR Treated**	**Medium Sterility CTRL**	**CAR Sterility CTRL**
	**0,9526**	**0,2969**	**0,2062**	**0,1986**
	**0,9861**	**0,1836**	**0,2681**	**0,246**
	**0,9518**	**0,1663**	**0,2437**	**0,2462**
	**0,8247**	**0,1645**	**0,2603**	**0,2406**
	**0,7595**	**0,1848**	**0,2482**	**0,1627**
	**0,7277**	**0,1664**	**0,2385**	**0,2361**
	**0,9844**	0,2243	**0,1764**	**0,1627**
	**0,9453**	**0,2743**	**0,2441**	**0,1555**
	**0,9797**	**0,1724**	**0,1764**	**0,1627**
	**0,9356**	**0,1916**	**0,2441**	**0,1555**

	**Positive CTRL**	**CAR Treated**	**Medium Sterility CTRL**	**CAR Sterility CTRL**
**Mean**	**0,90474**	**0,20251**	**0,2306**	**0,19666**
**subtraction of whites**	**0,67414**	**0,00585**		
	**Positive CTRL**	**CAR Treated**		

% Biofilm formation	100	0,867772273

AlamarBlue assay for MBIC determination of **carvacrol (**CAR**)** *versus Helicobacter pylori*

**First experiment**	**Positive CTRL**	**CAR Treated**	**Medium Sterility CTRL**	**CAR Sterility CTRL**
570 nm	**0,423**	**0,2863**	**0,3491**	**0,3404**
	**0,4491**	**0,3105**	**0,3376**	**0,3453**
	**0,4594**	**0,3462**	**0,3366**	**0,3378**
	**0,4646**	**0,3539**	**0,3352**	**0,3221**

	**Positive CTRL**	**CAR Treated**	**Medium Sterility CTRL**	**CAR Sterility CTRL**
670 nm	**0,2101**	**0,3294**	**0,3914**	**0,3861**
	**0,202**	**0,3538**	**0,3816**	**0,389**
	**0,2072**	**0,3933**	**0,3824**	**0,3809**
	**0,2305**	**0,4041**	**0,3806**	**0,3695**

	**Positive CTRL**	**CAR Treated**	**Medium Sterility CTRL**	**CAR Sterility CTRL**
**(570 nm)**	**0,449025**	**0,324225**	**0,339625**	**0,3364**
**(600 nm)**	**0,21245**	**0,37015**	**0,384**	**0,381375**

	**Positive CTRL**	**CAR Treated**
**MBIC**	**64,79921108**	**15,01670066**

**Second experiment**	**Positive CTRL**	**CAR Treated**	**Medium Sterility CTRL**	**CAR Sterility CTRL**
570 nm	**0,4317**	**0,3092**	**0,207**	**0,3263**
	**0,3605**	**0,3299**	**0,3865**	**0,3146**
	**0,418**	**0,3081**	**0,3237**	**0,2925**
	**0,3749**	**0,3094**	**0,3261**	**0,2694**

	**Positive CTRL**	**CAR Treated**	**Medium Sterility CTRL**	**CAR Sterility CTRL**
600 nm	**0,1924**	**0,3581**	**0,2325**	**0,3681**
	**0,1804**	**0,3625**	**0,3129**	**0,3529**
	**0,2067**	**0,3093**	**0,3621**	**0,3293**
	**0,1844**	**0,3278**	**0,3665**	**0,3078**

	Positive CTRL	CAR Treated	Medium Sterility CTRL	CAR Sterility CTRL
**(570 nm)**	**0,39628**	**0,31415**	**0,31083**	**0,30070**
**(600 nm)**	**0,19098**	**0,33943**	**0,31850**	**0,33953**

	Positive CTRL	CAR Treated
**MBIC**	**68,97758462**	**19,54681043**

CFU count for MBIC determination of **carvacrol (**CAR**)** *versus Helicobacter pylori*

	Non treated (CFU/mL)	CAR Treated (CFU/mL)
**First experiment**	**4,43E+07**	**0,00E+00**
	**2,49E+07**	**0,00E+00**
**Second experiment**	**6,60E+07**	**0,00E+00**
	**6,20E+07**	**0,00E+00**

Cristal violet data for MBIC determination of **thymol (**THY**)** *versus Helicobacter pylori*

First experiment	Positive CTRL	THY Treated	Medium Sterility CTRL	THY Sterility CTRL
	1,5161	0,8537	0,5647	0,6769
	1,5774	0,7529	0,4973	0,5269
	1,3881	0,8514	0,5434	0,7480
	1,5479	0,9176	0,5647	0,8963
	1,5107	0,6881	0,7548	0,7868
	2,5187	0,7480	0,5647	0,8918
	2,0421	0,707	0,8682	0,7794
	2,0092	0,8207	0,7548	0,8519
	1,8331	0,7771	0,4973	0,6769
	1,8954	0,7214	0,5434	0,5269

	Positive CTRL	THY Treated	Medium Sterility CTRL	THY Sterility CTRL
**Mean**	1,7839	0,7838	0,6153	0,7362
**subtraction of whites**	1,1685	0,0476		
	**Positive CTRL**	**thy Treated**		
**% Biofilm formation**	100	4,074314957		

**Second experiment**	Positive CTRL	THY Treated	Medium Sterility CTRL	THY Sterility CTRL
	2,0217	0,9475	0,7794	0,7794
	1,7013	1,0887	0,8049	1,1133
	1,6374	1,0115	0,8835	0,7882
	1,9662	1,4667	1,2155	0,9052
	1,6075	0,9475	0,8835	1,1533
	1,9513	0,7485	0,8049	0,9052
	2,4107	1,0115	0,8049	0,8049
	1,8531	0,7956	0,8835	0,7882
	1,5566	0,7625	1,2155	0,9052
	1,3254	0,8056	0,8049	1,1533

	Positive CTRL	THY Treated	Medium Sterility CTRL	THY Sterility CTRL
**Mean**	1,8031	0,9586	0,9081	0,9296
**subtraction of whites**	0,8951	0,0289		
	**Positive CTRL**	**TIM Treated**		

**% Biofilm formation**	**100**	**3,233266672**

AlamarBlue data for MBIC determination of **thymol** (THY) *versus Helicobacter pylori*

**First experiment**	**Positive CTRL**	**THY Treated**	**Medium Sterility CTRL**	**THY Sterility CTRL**
570 nm	**0,413**	**0,32**	**0,2269**	**0,3004**
	**0,418**	**0,3036**	**0,302**	**0,306**
	**0,3876**	**0,2837**	**0,2859**	**0,3121**
	**0,4183**	**0,2917**	**0,2889**	**0,2988**

	**Positive CTRL**	**THY Treated**	**Medium Sterility CTRL**	**THY Sterility CTRL**
600 nm	**0,1349**	**0,3755**	**0,2664**	**0,3592**
	**0,1988**	**0,3575**	**0,3599**	**0,3682**
	**0,1884**	**0,3318**	**0,3377**	**0,37940**
	**0,1444**	**0,3439**	**0,346**	**0,35900**

	**Positive CTRL**	**THY Treated**	**Medium Sterility CTRL**	**THY Sterility CTRL**
**(570 nm)**	**0,4092**	**0,2998**	**0,2759**	**0,3043**
**(600 nm)**	**0,166625**	**0,352175**	**0,3275**	**0,36645**

	**Positive CTRL**	**THY Treated**
**MBIC**	**73,58131957**	**12,84515501**

**Second experiment**	**Positive CTRL**	**THY Treated**	**Medium Sterility CTRL**	**THY Sterility CTRL**
570 nm	**0,4207**	**0,3007**	**0,3068**	**0,3137**
	**0,45**	**0,3259**	**0,2743**	**0,3435**
	**0,4369**	**0,2767**	**0,2958**	**0,2983**
	**0,455**	**0,3319**	**0,2894**	**0,2822**

	**Positive CTRL**	**THY Treated**	**Medium Sterility CTRL**	**THY Sterility CTRL**
600 nm	**0,1602**	**0,3642**	**0,3713**	**0,3843**
	**0,1746**	**0,398**	**0,3345**	**0,4295**
	**0,1722**	**0,3386**	**0,3542**	**0,3661**
	**0,1664**	**0,4153**	**0,3477**	**0,342**

	Positive CTRL	THY Treated	Medium Sterility CTRL	THY Sterility CTRL
**(570 nm)**	**0,4407**	**0,3088**	**0,2916**	**0,3094**
**(600 nm)**	**0,16835**	**0,379025**	**0,351925**	**0,380475**

	Positive CTRL	THY Treated
**MBIC**	**75,39339975**	**10,33354534**

CFU count for MBIC determination of **thymol (**TIM**)** *versus Helicobacter pylori*

	Non treated (CFU/mL)	THY Treated (CFU/mL)
**First experiment**	**1,46E+07**	**0,00E+00**
	**2,97E+07**	**0,00E+00**
**Second experiment**	**6,47E+07**	**0,00E+00**
	**5,33E+07**	**0,00E+00**

Flow cytometry analyses:

OMV concentrations obtained by flow cytometry analyses.

**Carvacrol**			
**Planktonic CTRL**		**Planktonic Carvacrol**	
Total OMVs	***OMVs eDNA+***	***Total OMVs***	***OMVs eDNA+***
45.99	15.00	16.50	9.00
70.80	33.00	11.01	1.50
44.40	15.00	10.20	3.00

**Thymol**			
**Planktonic CTRL**		**Planktonic Thymol**	
Total OMVs	***OMVs eDNA+***	***Total OMVs***	***OMVs eDNA+***
248.25	209.25	60.60	3.60
212.40	139.80	38.25	3.75
232.20	162.00	20.49	3.99
163.50	65.25	115.80	58.20
111.99	65.01	48.75	31.50
90.51	52.50	47.01	29.49

**Amoxicillin**			
**Planktonic CTRL**		**Planktonic Amoxicillin**	
Total OMVs	***OMVs eDNA+***	***Total OMVs***	***OMVs eDNA+***
33.60	10.80	36.00	7.80
39.00	21.75	28.00	7.80
40.50	18.75	26.75	6.75
58.14	8.25	31.26	1.38
60.36	9.75	48.00	5.13
67.80	9.60	36.87	3.42
**Biofilm CTRL**		**Biofilm Amoxicillin**	
Total OMVs	***OMVs eDNA+***	***Total OMVs***	***OMVs eDNA+***
527.40	372.60	510.75	225.00
630.00	447.75	450.99	225.51
568.20	393.60	593.40	331.20
142.75	29.36	162.66	26.67
218.66	108.00	209.64	51.75
287.29	147.58	238.86	61.14
